# Organic fertilizer improved the lead and cadmium metal tolerance of *Eucalyptus camaldulensis* by enhancing the uptake of potassium, phosphorus, and calcium

**DOI:** 10.3389/fpls.2024.1444227

**Published:** 2024-09-23

**Authors:** Linnan Ouyang, Shaoxiong Chen, Wentao Yang, Jiaqi Zheng, Lingshuai Ye, Qiang Liu, Jiaqi Yang

**Affiliations:** ^1^ Research Institute of Fast-growing Trees, Chinese Academy of Forestry, State Key Laboratory of Efficient Production of Forest Resources, Zhanjiang, China; ^2^ Key Laboratory of Karst Geological Resources and Environment, Ministry of Education, College of Resource and Environmental Engineering, Guizhou University, Guiyang, China; ^3^ College of Life and Environment Science, Central South University of Forestry and Technology, Changsha, China

**Keywords:** *Eucalyptus camaldulensis*, *Bacillus subtilis*, cultivation measures, heavy metal pollution, phytoremediation

## Abstract

Phytoremediation is a strategy for the amelioration of soil heavy metal contamination that aligns with ecological sustainability principles. Among the spectrum of phytoremediation candidates, woody plants are considered particularly adept for their substantial biomass, profound root systems, and non-participation in the food chain. This study used *Eucalyptus camaldulensis*—a tree species characterized for its high biomass and rapid growth rate—to assess its growth and metal uptake in mining tailings. The results were as follows: exposure to heavy metals reduced the *E. camaldulensis* uptake of potassium (K), phosphorus (P), and calcium (Ca). Heavy metal stress negatively affected the biomass of *E. camaldulensis*. Lead (Pb) primarily accumulated in the roots, while cadmium (Cd) predominantly accumulated in the stems. The application of organic fertilizers bolstered the stress tolerance of *E. camaldulensis*, mitigating the adverse impacts of heavy metal stress. A synergistic effect occurred when organic fertilizers were combined with bacterial fertilizers. The plant’s enrichment capacity for Cd and its tolerance to Pb was augmented through the concurrent application of bacterial and organic fertilizers. Collectively, the application of organic fertilizers improved the heavy metal tolerance of *E. camaldulensis* by enhancing the uptake of K, P, and Ca and elevating the content of glutathione peroxidase (GPX) and gibberellin acid (GA) in roots. These findings provided nascent groundwork for breeding *E. camaldulensis* with enhanced heavy metal tolerance. Moreover, this proved the potentiality of *E. camaldulensis* for the management of heavy metal-contaminated tailings and offers a promising avenue for future environmental restoration.

## 1 Introduction

Heavy metal stress, predominantly elicited by cadmium (Cd) and lead (Pb) (Di Toppi and Gabbrielli, 1999), represents an abiotic stressor that significantly impedes plant growth. Occurrence of heavy metal stress arises with the increase of mining and misuse of pesticides. Heavy metal contamination poses a critical threat to the sustainability and quality of agriculture (DalCorso et al., 2013; Zhou et al., 2020). The uptake of Cd and Pb by plants predominantly occurs in roots and leaves and induces toxic effects with high concentrations (White and Brown, 2010). Animals with prolonged exposure to environments contaminated with Cd and Pb suffer from a spectrum of diseases (Nawrot et al., 2006; Youness et al., 2012). In soil ecosystems, Cd and Pb are commonly present in the form of divalent cations and simple coordination complexes that diffuse easily and are difficult to contain (Ghori et al., 2016; Ma et al., 2014). As a result, the remediation of heavy metal-contaminated soils has become an urgent and enduring challenge that requires immediate and sustained attention.

Innovative phytoremediation has garnered considerable attention as an eco-friendly and low-cost approach (Ghori et al., 2016; Mahar et al., 2016). The research on phytoremediation so far has predominantly focused on herbaceous hyperaccumulators (Shutcha et al., 2010). However, employment of herbaceous plants for heavy metal soil management gains marginal remediation effect for limited metal uptake and low stress tolerance (Jing et al., 2023; Talebzadeh, 2023). In contrast, woody plants are more promising candidates for phytoremediation because of their extensive root systems, substantial biomass, and protracted life spans (Liu et al., 2009). *Eucalyptus* species were introduced from Australia and massively cultivated in South China for their rapid growth and substantial yield (Yu et al., 2019). Prior studies have proved their capacity to absorb heavy metal ions from the soil (Unterbrunner et al., 2007), with *Eucalyptus camaldulensis* exhibiting better stress tolerance (Rahman et al., 2022; Zhou et al., 2022).

Current research on alleviating heavy metal pollution by woody plants mostly focuses on the effects of individual elements (Liu et al., 2022; Marmiroli et al., 2011; Sahito et al., 2023; Song et al., 2024) and overlooks the complex soil conditions typically found in mining areas (Yang et al., 2022; Zhang Y. et al., 2023). To improve phytoremediation strategies, it is essential to investigate the heavy metal absorption capacity and physiological responses of woody plants to stress under mining conditions. In our study, we employed mining tailings as the stressor. We explored the physiological response of *E. camaldulensis* and heavy metal uptake under various amendments. This research endeavors to provide a theoretical framework and practical insights into the potential of *E. camaldulensis* in the bio-remediation of soils contaminated with heavy metals.

## 2 Materials and methods

### 2.1 Experimental design

The basic soil was taken from the *Eucalyptus* plantation in the National Forest Seedling Demonstration Base (110.15°E, 21.43°N), south of Zhanjiang City, Guangdong Province. The physicochemical properties of the tailings and organic fertilizer are shown in **Table 1**. The original pH of the tailings was 2.35. A total of 0.2% lime was added to the tailings uniformly and mixed to ensure the primary growth of plant seedlings. The three amendments used in the experiment were organic fertilizer, bacterial agent, and inorganic fertilizer. Organic fertilizer consists of poultry manure, straw compost, and peat soil. The bacterial agent is the dominant tolerance strain of *Bacillus subtilis*, which was screened from tailing separation and purified in the early studies. The strain had shown strong tolerance and removal ability of Cd and Pb (Yang et al., 2024). Inorganic fertilizer was purchased from Shanghai Yara Trading Co., Ltd. This compound fertilizer contains ammonium N, P_2_O_5_, and K_2_SO_4_. The total amounts of N, P, and K accounted for 13%, 6%, and 21%, respectively, of the fertilizer quality. The seedlings of *E. camaldulensis* (seed lot no. 20654, 30 cm tall) were provided by the Research Institute of Fast-growing Trees in the Chinese Academy of Forestry.

**Table 1:** Basic soil physical and chemical properties of the tailings and organic manure.

	pH	CEC (cmol/kg)	OM (g/kg)	T N (g/kg)	T P (g/kg)	T K (g/kg)	T Cd (mg/kg)	T Pb (mg/kg)
Mining tailings	2.35	4.23	2.06	0.17	0.85	2.32	1.83	2,025.99
Organic manure	9.02	34.77	641.92	15.2	7.69	21.3		
Cultivated land in China	4.50–9.10	<50	5.0–48	1.00–2.09	0.44–0.85	5.0–30.0		
China soil background values							0.1	26.0

CEC, matrix cation exchange capacity; OM, organic Material; T, total.

The field site is located in the National Forest Seedling Demonstration Base (110.15°E, 21.43°N), south of Zhanjiang City, Guangdong Province. Plastic pots with an inner diameter of 270 mm and a height of 225 mm were used to contain 4 kg of tailings each. Three amendment treatments and two matrices were set up (**Table 2**): Tailings + *E. camaldulensis* (K-C), Tailings + Inorganic Fertilizer + *E. camaldulensis* (K-W-C), Tailings + Organic Fertilizer + *E. camaldulensis* (K-Y-C), Tailings + Organic Fertilizer + *B. subtilis* + *E. camaldulensis* (K-Y-J-C), Tailings + Inorganic Fertilizer + *B. subtilis* + *E. camaldulensis* (K-W-J-C), Basic soil + *E. camaldulensis* (H-C), and Basic soil + Inorganic Fertilizer + *E. camaldulensis* (H-W-C). The single application of *B. subtilis* was excluded from the experiment, as *B. subtilis* showed no significant effect on plant growth under tailings in pre-trials (Yang et al., 2024). Organic fertilizer was added at a dose of 100 g/kg. The bacterial agent was added at 10 mL/kg under a concentration of 2 × 10^8^ *B. subtilis*/mL. Inorganic fertilizer was added at a dose of 12 g/kg. N, P, and K were added in equal amounts in the treatment groups of organic fertilizer and inorganic fertilizer. Each treatment had six replicates. Two *E. camaldulensis* were planted in each pot.

**Table 2:** Fertilizer treatments.

Situations tested	Treatment method
K-C	Mining tailings + *Eucalyptus camaldulensis*
K-W-C	Mining tailings + Inorganic Fertilizer + *E. camaldulensis*
K-Y-C	Mining tailings + Organic Fertilizer + *E. camaldulensis*
K-Y-J-C	Mining tailings + Organic Fertilizer + *Bacillus subtilis* + *E. camaldulensis*
K-W-J-C	Mining tailings + Inorganic Fertilizer + *B. subtilis* + *E. camaldulensis*
H-C	Basic soil + *E. camaldulensis*
H-W-C	Basic soil + Inorganic Fertilizer + *E. camaldulensis*

### 2.2 Measurement of plant phenotypic characteristics

Plant height and ground diameter were measured using tape measures and Vernier calipers. Afterward, all plants were removed from the soil with their roots. Fresh plants were immediately mixed and divided into two parts. The fresh weight of one part was measured before dehydrating in the oven (105°C for 30 minutes, followed by drying at 70°C to a constant weight). The samples were then weighed, crushed, sieved, bagged, and stored for further analysis. One part was stored at −80°C for further plant active ingredient analysis.

### 2.3 Measurement of plant element content

The Pb and Cd contents in the roots, stems, and leaves of *E. camaldulensis* were measured using an atomic absorption spectrometer (Contra AA 300) after digestion with a mixture of concentrated HNO_3_ and HClO_4_ (Narin et al., 2004). The contents of carbon (C), potassium (K), phosphorus (P), nitrogen (N), calcium (Ca), and magnesium (Mg) were measured using various analytical methods (Shan et al., 2024; Du et al., 2024; Jiang, 2023). Total N was measured using a diffusion method, and total P was measured using the vanadium molybdenum yellow colorimetry. The potassium chromate oxidation-capacity method was employed to measure total C. The flame laser meter method was used for K, and the inductively coupled plasma–optical emission spectrometry (ICP-OES) was applied to detect Ca and Mg.

### 2.4 Measurement of plant biochemical characteristics

The contents of enzymes [superoxide dismutase (SOD), catalase (CAT), peroxidase (POD), 1-aminocyclopropane-1-carboxylate deaminase (ACCD), and glutathione peroxidase (GPX)], malondialdehyde (MDA), metallothionein (MT), phytochelatins (PCSs), glutathione (GSH), chlorophyll, and hormones [gibberellins (GAs), indole acetic acid (IAA), cytokinin (CTK), and abscisic acid (ABA)] in leaves and roots were measured using a double-antibody sandwich enzyme-linked immunosorbent assay (ELISA) (Sofy et al., 2021). Siderophores of roots were measured using ferrous iron colorimetry methods (Li et al., 2024). Chopped plant tissue was ground into powder and mixed with homogenate (1 g of tissue with 9 mL of homogenate). The mixture was then centrifuged to obtain the supernatant, which was measured using the protocol provided by the kits (SOD, JN6208; CAT, JN83958; POD, N08981; MDA, N823562; ACCD, JN04449; GPX, JN840181; MT, JN846174; PCS, JN6282; GSH, JN82023; chlorophyll, JN09194; chlorophyll *a*, JN08953; chlorophyll *b*, JN09617; GA, JN6237; IAA, N09531; CTK, JN84212; ABA, JN09531; and siderophores, M1217L) (sourced from Shanghai Jining Shiye Co., Ltd., Shanghai, China). Reactive oxygen species (ROS) in leaves and roots were measured using the chemical fluorescence method and ROS detection reagent kit. The reagents were sourced from Shanghai Jining Shiye Co., Ltd., and the measurements were conducted following the JN872201 kit’s protocol.

The contents of root exudates, specifically oxalic acid (OA) and citric acid (CA), were analyzed in liquid chromatography (Javed et al., 2017). One gram of the sample was ground with liquid nitrogen and mixed with a pre-chilled 20-mL solution of 0.1% phosphoric acid. The mixture was agitated for 10 minutes and subjected to ultrasonic oscillation at a low temperature for 40 minutes. The extraction solution was then adjusted to 28 mL, agitated for 5 minutes, and centrifuged at 4,000 rpm for 15 minutes. Samples (20 μL for each) were then analyzed using an Agilent 1200 high-efficiency liquid chromatography analyzer equipped with a TSKgel ODS-100V column (4.6 mm × 250 mm × 5.0 μm). A 0.1% phosphate solution served as the mobile phase, with detection at a wavelength of 210 nm and column temperature of 40°C.

### 2.5 Measurement of matrix physicochemical properties

The pH was measured using an acidimeter (PHS-3C, Leici, Shanghai, China). Organic matter was measured using the potassium dichromate oxidation-spectrophotometric method as described by Nelson and Sommers (1996). The cation exchange capacity (CEC) was measured using the barium chloride–sulfuric acid forced exchange method, following the protocol by Meier and Kahr (1999).

### 2.6 Data analysis

Statistical analysis of data was conducted using a combination of Microsoft Excel 2010 and R 4.2.2. The “Rmisc”, “ggplot2”, “agricolae” and “psych” packages in R were used to calculate the ANOVA, Duncan multiple comparisons, and correlation coefficients. Visualization was conducted using GraphPad Prism 9 and ChiPlot (www.chiplot.online).

## 3 Results

### 3.1 Inter-sample correlation analysis of fertilizer treatments

**Figure 1:**
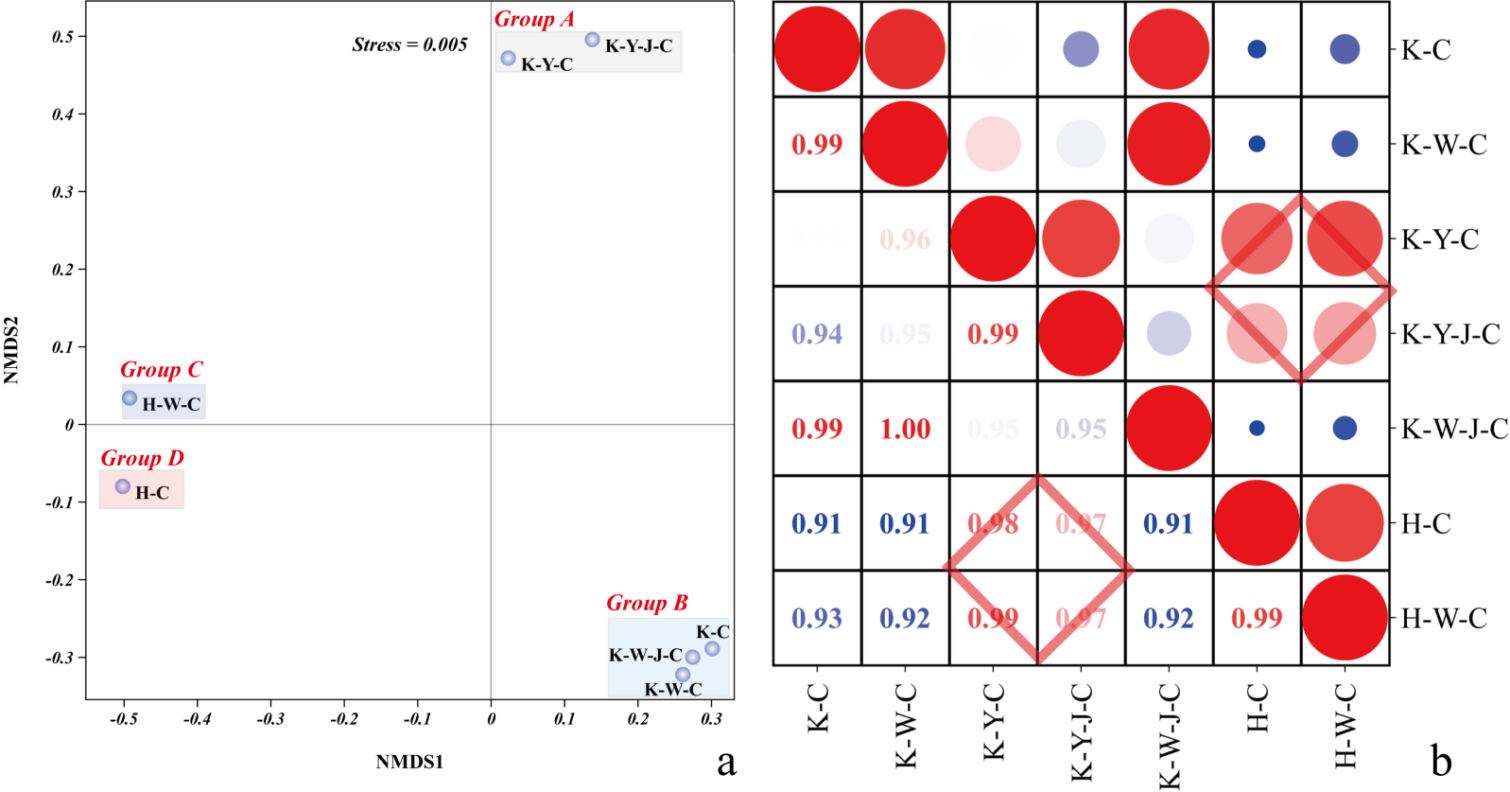
Analysis of treatment intergroup correlation. **(A)** Non-metric multidimensional scaling analysis. **(B)** Correlation analysis between sample groups. Upper portion is sample correlation analysis picture, and bottom part is correlation coefficient. Red, big circles indicate stronger relationships, and blue small circles are weaker ones. Pearson’s r on R calculated correlations. Mining tailings + *Eucalyptus camaldulensis* (K-C), Mining tailings + Inorganic Fertilizer + *E. camaldulensis* (K-W-C), Mining tailings + Organic Fertilizer + *E. camaldulensis* (K-Y-C), Mining tailings + Organic Fertilizer + *Bacillus subtilis* + *E. camaldulensis* (K-Y-J-C), Mining tailings + Inorganic Fertilizer + *B. subtilis* + *E. camaldulensis* (K-W-J-C), Basic soil + *E. camaldulensis* (H-C), and Basic soil + Inorganic Fertilizer + *E. camaldulensis* (H-W-C).

Non-metric multidimensional scaling (NMDS) analyses were conducted on all measurements (plant phenotypic characteristics, element content, plant biochemical characteristics, and matrix physicochemical properties) (**Figure 1A**). The result showed pronounced divergences of the *E. camaldulensis* growth between the mining tailing treatments and normal soil treatments along NMDS1. The treatments were discernibly separated into four distinct groups in the NMDS1 and NMDS2 biplots. Group A underscored the significant mitigating effect of organic fertilizer in mining tailings. Conversely, Group B showed a negligible effect of inorganic fertilizer on the plant developmental trajectory under stress conditions. Correlational analysis was executed using the aforementioned dataset (**Figure 1B**). A comparative assessment among the quintet of treatment groups—Basic soil + *E. camaldulensis* (H-C), Inorganic Fertilizer + *E. camaldulensis* (H-W-C), Mining tailings + Organic Fertilizer + *E. camaldulensis* (K-Y-C), Mining tailings + Organic Fertilizer + *B. subtilis* + *E. camaldulensis* (K-Y-J-C), and Mining tailings + *E. camaldulensis* (K-C)—showed that the application of organic fertilizer, alone and combined with bacterial fertilizers, significantly bolstered the resilience of *E. camaldulensis* to heavy metal stress.

### 3.2 The effect of fertilizer treatments on the phenotypic traits in *E. camaldulensis*

**Figure 2:**
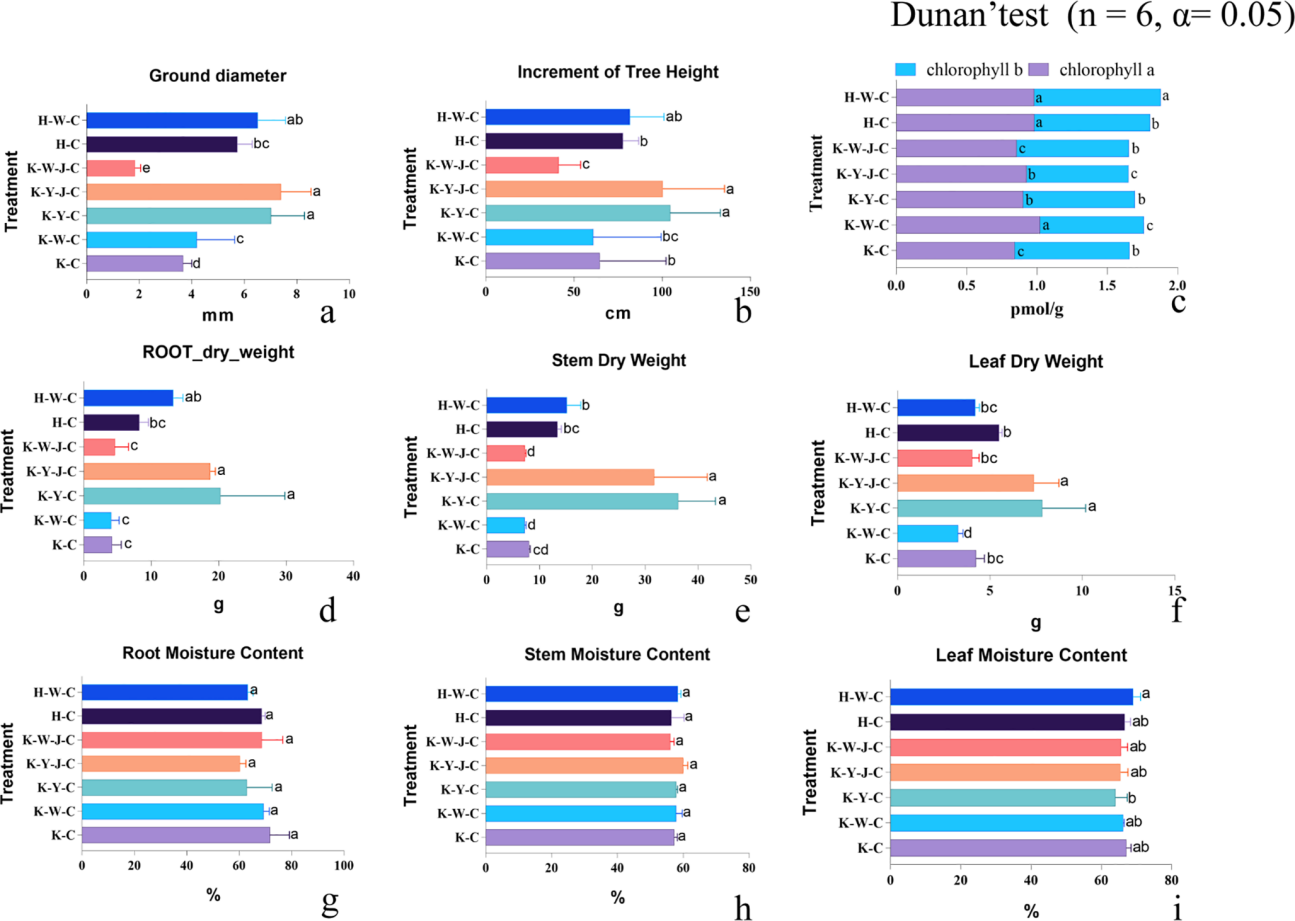
Treatment-group phenotypes. **(A)** Ground diameter. **(B)** Height growth. **(C)** Chlorophyll content. **(D)** Root dry weight. **(E)** Stem dry weight. **(F)** Leaf dry weight. **(G)** Root moisture content. **(H)** Stem moisture content. **(I)** Leaf moisture content. Mining tailings + *Eucalyptus camaldulensis* (K-C), Mining tailings + Inorganic Fertilizer + *E. camaldulensis* (K-W-C), Mining tailings + Organic Fertilizer + *E. camaldulensis* (K-Y-C), Mining tailings + Organic Fertilizer + *Bacillus subtilis* + *E. camaldulensis* (K-Y-J-C), Mining tailings + Inorganic Fertilizer + *B. subtilis* + *E. camaldulensis* (K-W-J-C), Basic soil + *E. camaldulensis* (H-C), and Basic soil + Inorganic Fertilizer + *E. camaldulensis* (H-W-C).

The study commenced on September 1, 2020, and data collection was concluded on May 1, 2022. Analysis of ground diameter growth (**Figure 2A**) revealed that the mining tailings caused a diameter reduction of 56.3%. Inorganic fertilizers had no significant impact on growth under basic soil conditions, as observed in treatments H-W-C and H-C. However, under conditions of mining tailings, inorganic fertilizers potentially mitigate the adverse effects as ground diameter increased by 14.12% in fertilized soil (K-W-C and K-C). Contrarily, the combined application of inorganic and bacterial fertilizers (K-W-J-C) seemed to intensify heavy metal stress and resulted in a significant decrease of ground diameter by 56.01% compared to a single application of inorganic fertilizer (K-W-C). Notably, no significant disparity in ground diameter was detected among the treatment groups K-Y-C, K-Y-J-C, and H-W-C. In general, compared to the H-C treatment group, organic fertilizers effectively counterbalanced the stress from mining tailings and increased ground diameter by 22.14%, 28.77%, and 13.57% for K-Y-C, K-Y-J-C, and H-W-C, respectively.

Regarding tree height depicted in **Figure 2B**, mining tailings did not exert a significant effect on height in treatments H-C and K-C. Inorganic fertilizers had negligible effect on increasing height growth under basic soil conditions (H-W-C and H-C). However, application of inorganic fertilizer ameliorated the stress effect of mining tailings (H-W-C and K-W-C). The utilization of organic fertilizers enhanced tree height under mining tailings conditions. Under conditions of mining tailings, the height was 61.61% and 55.12% higher for organic fertilizer treated (K-Y-C) and organic-bacteria fertilizer treated (K-Y-J-C), respectively, than unfertilized (K-C). The tree height was 34.39% and 28.99% higher for organic fertilizer treated (K-Y-C) and organic-bacteria fertilizer treated on mining tailings (K-Y-J-C), respectively, than unfertilized on basic soil (H-C).

Inorganic fertilizers applied to basic soil (H-W-C) increased chlorophyll *b* levels (**Figure 2C**). Mining tailings (K-C) stress significantly impacted the accumulation of chlorophyll *a* (K-C and H-C). Under conditions of mining tailings, inorganic fertilizers (K-W-C) significantly elevated both chlorophyll *a* and *b* levels. Organic fertilizers applied to mining tailings significantly increased chlorophyll *a* content (K-C and K-Y-C). Moreover, the combined application of organic and bacterial fertilizers to mining tailings significantly increased chlorophyll *a* level and reduced chlorophyll *b*. In contrast, the combined application of inorganic fertilizer and bacterial fertilizer (K-W-J-C) to mining tailings had no significant effect on chlorophyll content. However, compared to a single application of inorganic fertilizer (K-W-C), the application of bacterial fertilizer in addition to inorganic fertilizers (K-W-J-C) significantly reduced the content of chlorophyll *a* and increased the content of chlorophyll *b*.

Lastly, the dry weight of roots and stems had no substantial effect on the dry weight of leaves, roots, and stems (treatments H-C and K-C) (**Figures 2D–F**). The application of inorganic fertilizers to basic soil (H-W-C) had no effect on the dry weight of all plant parts. Inorganic fertilizers—applied alone (K-W-C) or with bacteria fertilizer (K-W-J-C)—did not increase the dry weight of any plant parts. Under conditions of mining tailings, the single application of inorganic fertilizer (K-W-C) even reduced leaf dry weight (**Figure 2F**). On the contrary, plants on mining tailings gained more dry weight than under unfertilized basic soil (H-C) when supplied with organic fertilizer alone (K-Y-C) and, in combination with bacterial fertilizers (K-Y-J-C), significantly increased the dry weight of all plant parts. Under conditions of mining tailings, the increase of dry weight by organic fertilizers (K-Y-C) was 385.30% for roots and 348.96% with bacterial fertilizers (K-Y-J-C). For stems, the increase was 349.79% alone (K-Y-C) and 293.50% with bacterial fertilizers (K-Y-J-C). For leaves, the increase was 84.52% alone (K-Y-C) and 73.92% with bacterial fertilizers (K-Y-J-C). No effects of treatment on moisture content were found (**Figures 2G–I**).

### 3.3 The effect of fertilizer treatments on the physiological response in *E. camaldulensis*

**Figure 3:**
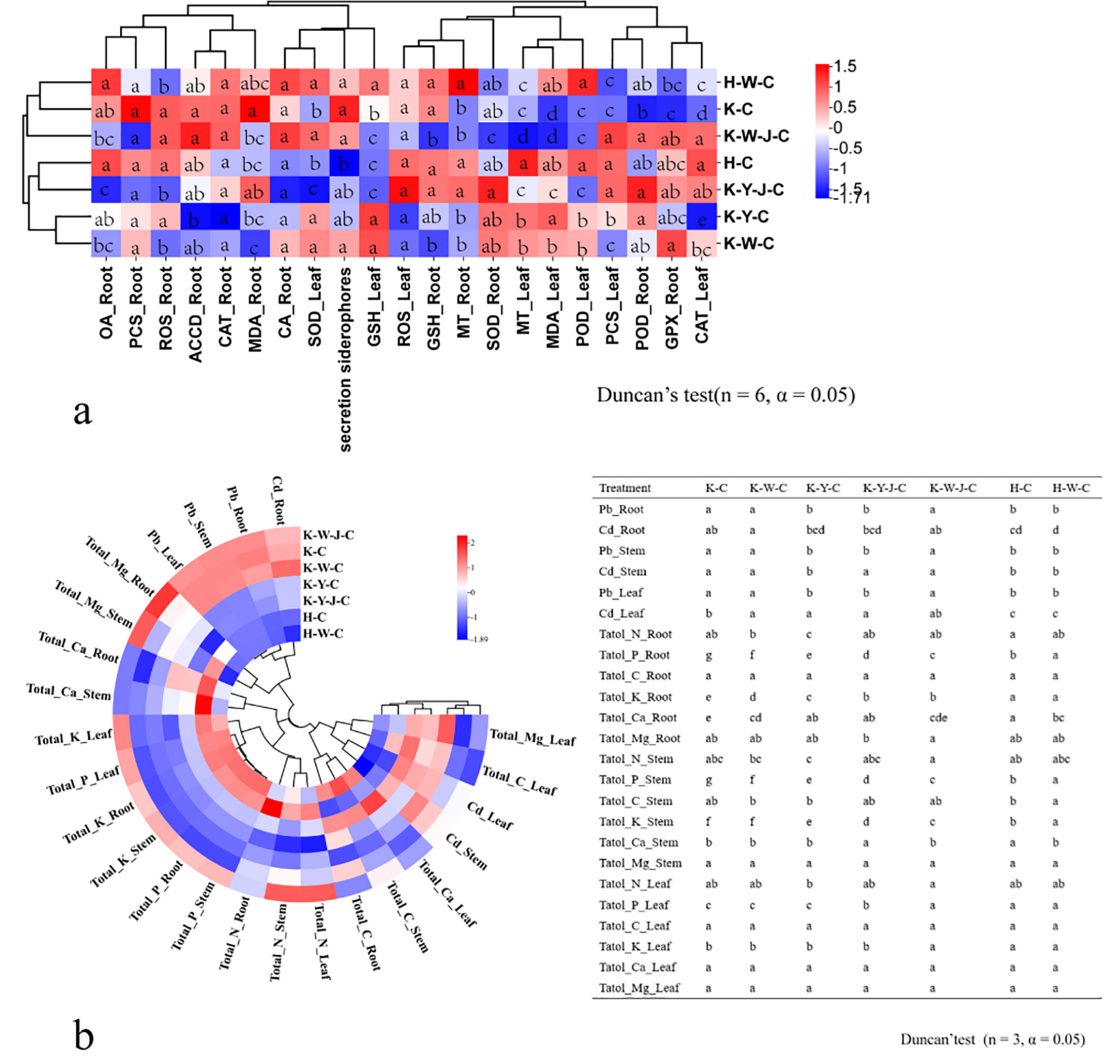
Differences in physiological indicators and elements. **(A)** Changes in stress physiological indicators of roots, stems, and leaves. **(B)** Changes in elements in roots, stems, and leaves. Mining tailings + *Eucalyptus camaldulensis* (K-C), Mining tailings + Inorganic Fertilizer + *E. camaldulensis* (K-W-C), Mining tailings + Organic Fertilizer + *E. camaldulensis* (K-Y-C), Mining tailings + Organic Fertilizer + *Bacillus subtilis* + *E. camaldulensis* (K-Y-J-C), Mining tailings + Inorganic Fertilizer + *B. subtilis* + *E. camaldulensis* (K-W-J-C), Basic soil + *E. camaldulensis* (H-C), and Basic soil + Inorganic Fertilizer + *E. camaldulensis* (H-W-C). Superoxide dismutase (SOD), catalase (CAT), peroxidase (POD), malondialdehyde (MDA), 1-aminocyclopropane-1-carboxylate deaminase (ACCD), glutathione peroxidase (GPX), metallothionein (MT), phytochelatins (PCSs), glutathione (GSH), oxalic acid (OA), and citric acid (CA).

**Figure 4:**
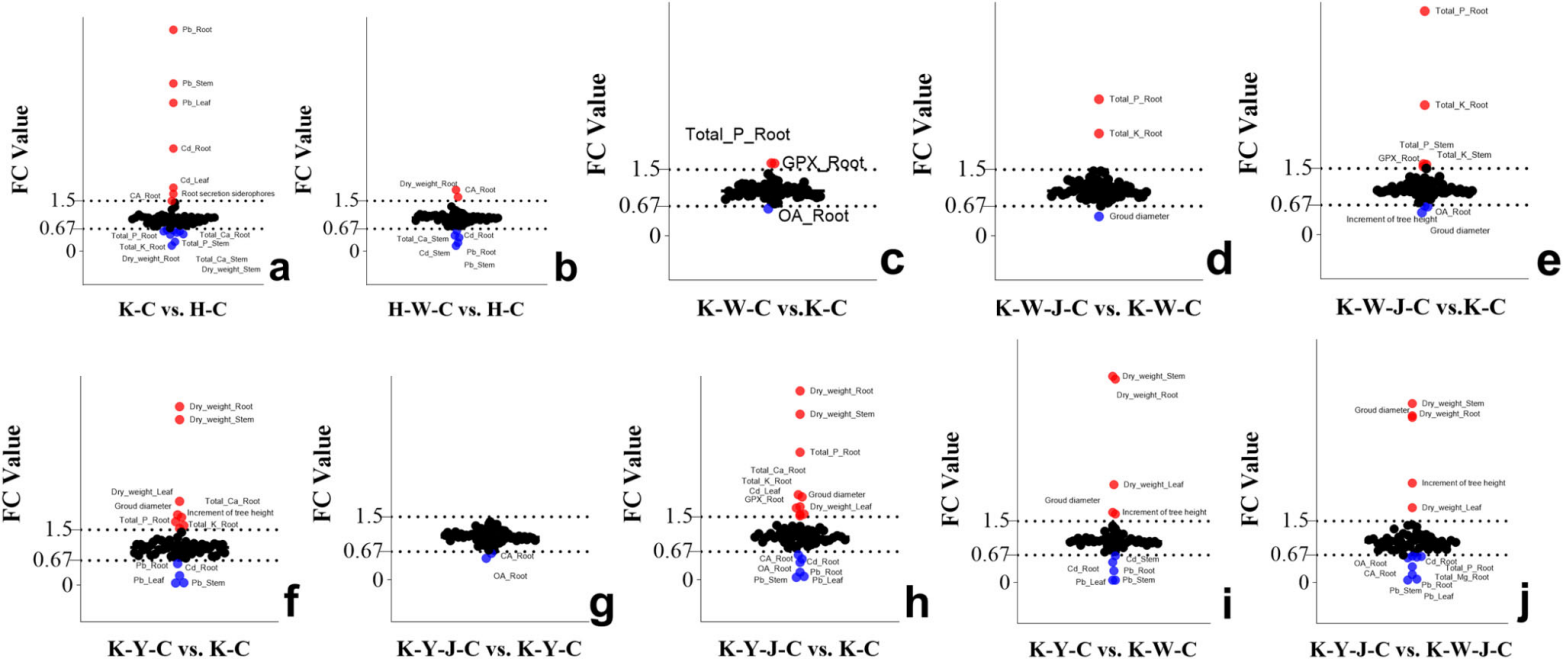
Analysis of phenotypic differences between groups. **(A)** (FC value of K-C vs. H-C), **(B)** (FC value of H-W-C vs. H-C), **(C)** (FC value of K-W-C vs. K-C), **(D)** (FC value of K-W-J-C vs. K-W-C), **(E)** (FC value of K-W-J-C vs. K-C), **(F)** (FC value of K-Y-C vs. K-C), **(G)** (FC value of K-Y-J-C vs. K-Y-C), **(H)** (FC value of K-Y-J-C vs. K-C), **(I)** (FC value of K-Y-C vs. K-W-C), **(J)** (FC value of K-Y-J-C vs. K-W-J-C). Screening criteria: FC > 1.5 and FC < 0.67. Mining tailings + *Eucalyptus camaldulensis* (K-C), Mining tailings + Inorganic Fertilizer + *E. camaldulensis* (K-W-C), Mining tailings + Organic Fertilizer + *E. camaldulensis* (K-Y-C), Mining tailings + Organic Fertilizer + *Bacillus subtilis* + *E. camaldulensis* (K-Y-J-C), Mining tailings + Inorganic Fertilizer + *B. subtilis* + *E. camaldulensis* (K-W-J-C), Basic soil + *E. camaldulensis* (H-C), and Basic soil + Inorganic Fertilizer + *E. camaldulensis* (H-W-C). SOD, Superoxid dismutase; CAT, Catalase; POD, Peroxidase; MDA, Malondialdehyde; ACCD, 1-aminocyclopropane-1-carboxylate deaminase; GPX, Glutathione peroxidase; MT, Metallothionein; PCS, Phytochelatins; GSH, Glutathione; OA, Oxalic acid; CA, Citric acid; _Root, the content of root; _Stem, the content of stem; _Leaf, the content of leaf.

Cluster analysis and differential analysis of physiological indices and elemental accumulation traits were conducted (**Figure 3**). Subsequently, a comparative differential analysis was undertaken between individual samples, employing stringent screening criteria that mandated a Fold Change (FC) exceeding 1.5 or falling below 0.67 (**Figure 4**).

Significant increase of Pb and Cd occurred in all parts of the plants with exposure to mining tailings (**Figure 4A**). The secretion of siderophores and CA significantly increased within the roots, while the contents of K, P, Ca, and biomass significantly reduced in both roots and stems (**Figure 4A**). The application of inorganic fertilizers under basic soil conditions significantly increased the CA content and dry weight of roots. On the contrary, the concentrations of Pb and Cd decreased in the roots and stems with inorganic fertilizers applied to basic soil. The Ca content also decreased in the stems with inorganic application to basic soil (**Figure 4B**). In the presence of mining tailings, inorganic fertilizers reduced OA levels and increased P and GPX within the roots (**Figure 4C**). The combined application of bacterial fertilizers and inorganic fertilizers has been shown to reduce the OA content in the roots and the growth of tree height and ground diameter. This combined treatment also led to an increase in the levels of K and P in the roots and stems and an increase in GPX activity in the roots (**Figure 4E**).

The combined application of bacterial fertilizer and inorganic fertilizer induced a substantial increase in the levels of P and K in the root system and reduced the ground diameter (**Figure 4D**). Under conditions of mining tailings, organic fertilizers significantly increased the ground diameter, tree height growth, and dry weight. They also increased the concentrations of Ca, P, and K in the roots. Additionally, they decreased the content of Pb in all plant parts and the content of Cd only in the roots (**Figure 4F**). The combined application of organic and bacterial fertilizers significantly reduced the contents of Cd, Pb, OA, and CA in the roots while significantly increasing P, K, Ca, and GPX in the roots and Cd in the leaves. This combined treatment also significantly increased the ground diameter and the dry weight of all plant parts (**Figure 4H**). The subsequent addition of bacterial fertilizer after organic fertilizer reduced the levels of OA and CA in the roots (**Figure 4G**).

Organic fertilizers, in contrast to inorganic fertilizers, significantly increased the ground diameter, tree height, and dry weight but reduced the concentrations of Cd and Pb in all plant parts (**Figure 4I**). In comparison to inorganic fertilizers, bacterial fertilizers combined with organic fertilizers significantly increased the tree height, ground diameter, and dry weight of all plant parts. The combined application of bacterial fertilizers and organic fertilizers also decreased OA, CA, Cd, P, and Mg contents in roots and Pb in all parts (**Figure 4J**).

### 3.4 The effect of fertilizer treatments on the hormone contents in *E. camaldulensis*

Under conditions of mining tailings, the concentration of GAs in the roots increased significantly. The combined application of organic and bacterial fertilizers significantly increased the concentration of GAs in leaves. The combined application of inorganic and bacterial fertilizers significantly increased CTKs in roots. On the contrary, the combination of organic and bacterial fertilizers significantly reduced the content of IAA. In terms of foliar response to mining tailings stress, an increase in GA content was found with bacterial fertilizer causing obvious enhancement. It is noteworthy that bacterial fertilizer application after organic fertilizer amplified this stimulatory effect. Inorganic fertilizer significantly decreased the IAA content in leaves. Across all treatment groups, there was a consistent and effective reduction in the levels of ABA in leaves.

### 3.5 Correlative analysis of hormonal and elemental responses in *E. camaldulensis* to mining tailings stress

**Figure 5:**
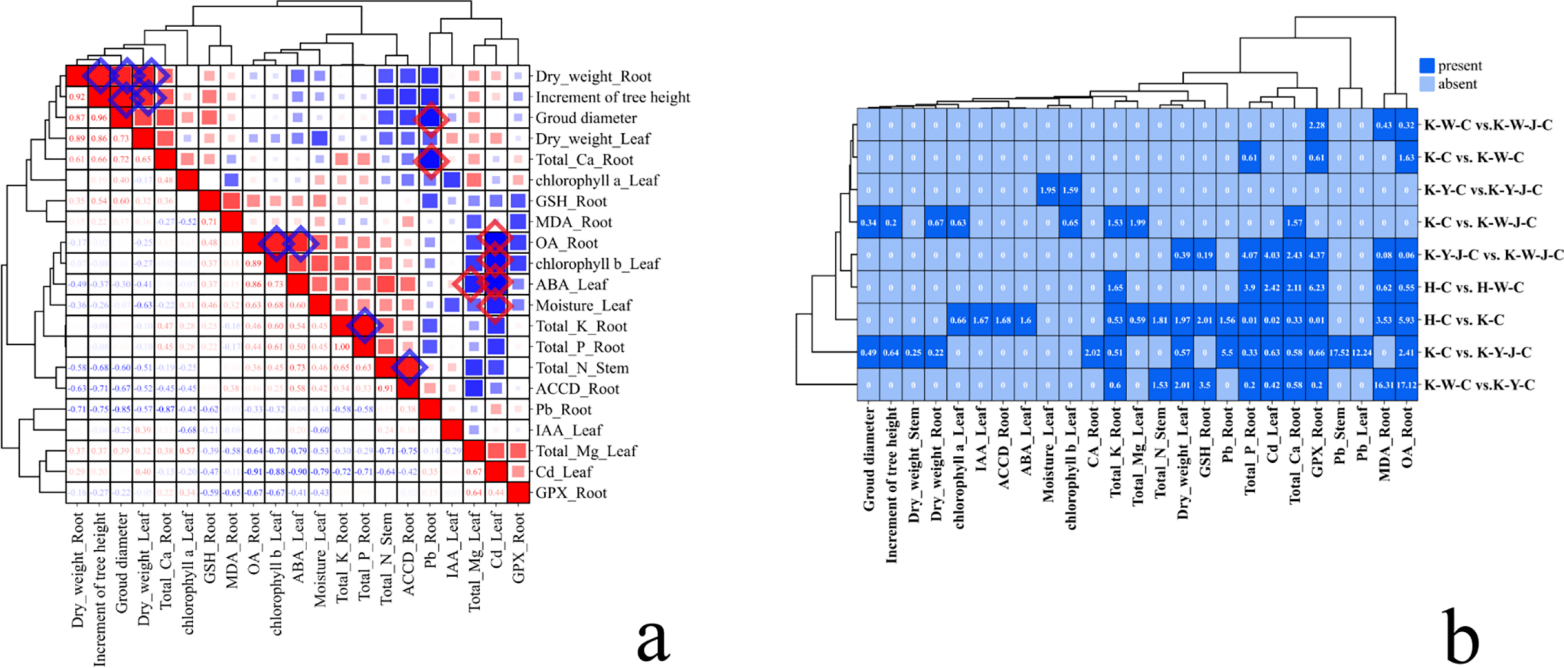
Correlative analysis of hormonal and elemental responses. **(A)** Correlation analysis between different materials. Red is positive correlation, and blue is negative correlation. **(B)** Binary plot of differential substances in roots, stems, and leaves between samples. Dark blue means there are differences in substances, and light blue means there are no differences in substances. Mining tailings + *Eucalyptus camaldulensis* (K-C), Mining tailings + Inorganic Fertilizer + *E. camaldulensis* (K-W-C), Mining tailings + Organic Fertilizer + *E. camaldulensis* (K-Y-C), Mining tailings + Organic Fertilizer + *Bacillus subtilis* + *E. camaldulensis* (K-Y-J-C), Mining tailings + Inorganic Fertilizer + *B. subtilis* + *E. camaldulensis* (K-W-J-C), Basic soil + *E. camaldulensis* (H-C), and Basic soil + Inorganic Fertilizer + *E. camaldulensis* (H-W-C). MDA, Malondialdehyde; ACCD, 1-aminocyclopropane-1-carboxylate deaminase; GPX, Glutathione peroxidase; GSH, Glutathione; GA, Gibberellins; IAA, Indole acetic acid; CTK, Cytokinin; ABA, Abscisic acid; OA, Oxalic acid; CA, Citric acid.

**Figure 6:**
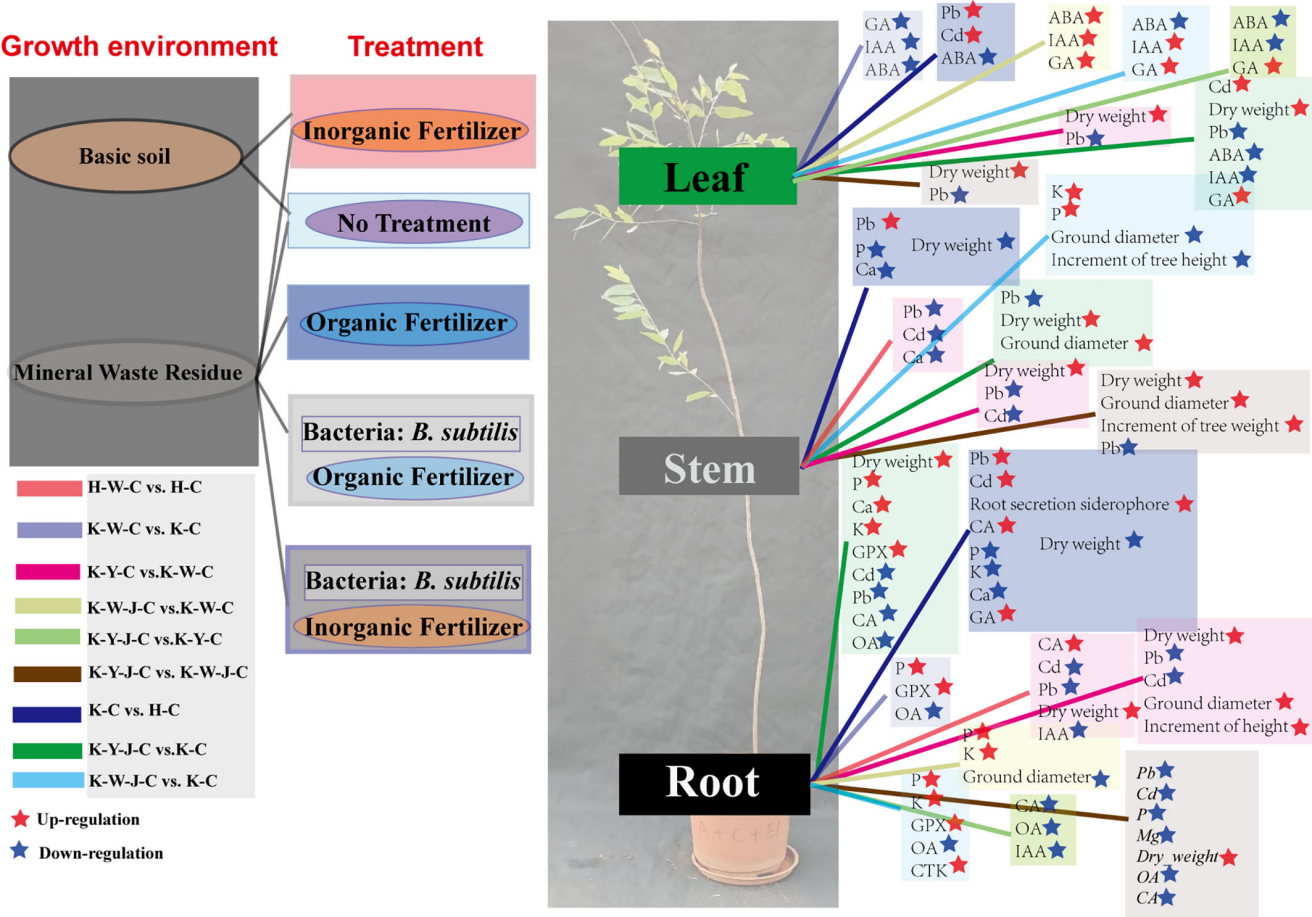
The schematic map of material and hormonal differences between fertilizer treatments. Mining tailings + *Eucalyptus camaldulensis* (K-C), Mining tailings + Inorganic Fertilizer + *E. camaldulensis* (K-W-C), Mining tailings + Organic Fertilizer + *E. camaldulensis* (K-Y-C), Mining tailings + Organic Fertilizer + *Bacillus subtilis* + *E. camaldulensis* (K-Y-J-C), Mining tailings + Inorganic Fertilizer + *B. subtilis* + *E. camaldulensis* (K-W-J-C), Basic soil + *E. camaldulensis* (H-C), and Basic soil + Inorganic Fertilizer + *E. camaldulensis* (H-W-C). GPX, Glutathione peroxidase; GA, Gibberellins; IAA, Indole acetic acid; CTK, cytokinin; ABA, Abscisic acid; OA, Oxalic acid; CA, Citric acid.

Comparative analysis was conducted to explore the change in hormone profiles by treatment (**Figures 5B**, **6**). Subsequently, a correlational analysis was executed (**Figure 5A**). Robust positive correlations among several parameters were found, including root dry weight, tree height growth, ground diameter, and leaf dry weight. Furthermore, the content of root OA positively correlated with the leaf’s chlorophyll *b* and ABA, suggesting a potential influence of root OA on the chlorophyll *b* accumulation and ABA synthesis within the leaves. In roots, K content positively correlated with P content. The stem N positively correlated with the root ACCD. Conversely, in roots, the Pb content negatively correlated with both Ca content and ground diameter.

In leaves, the ABA content negatively correlated with the Mg content. The leaf Cd content negatively correlated with the leaf water content, ABA, chlorophyll *b*, and root OA. This correlation suggests that Cd-induced stress in *E. camaldulensis* leaves is primarily reflected in the change of chlorophyll *b* and water content, with ABA potentially serving as a principal regulatory compound in the foliar response to Cd stress. Furthermore, the OA content in the roots may play a pivotal role in the translocation of Cd to the aerial parts of the plant.

### 3.6 The effect of fertilizer treatments on the absorption of Cd and Pb in *E. camaldulensis*

**Figure 7:**
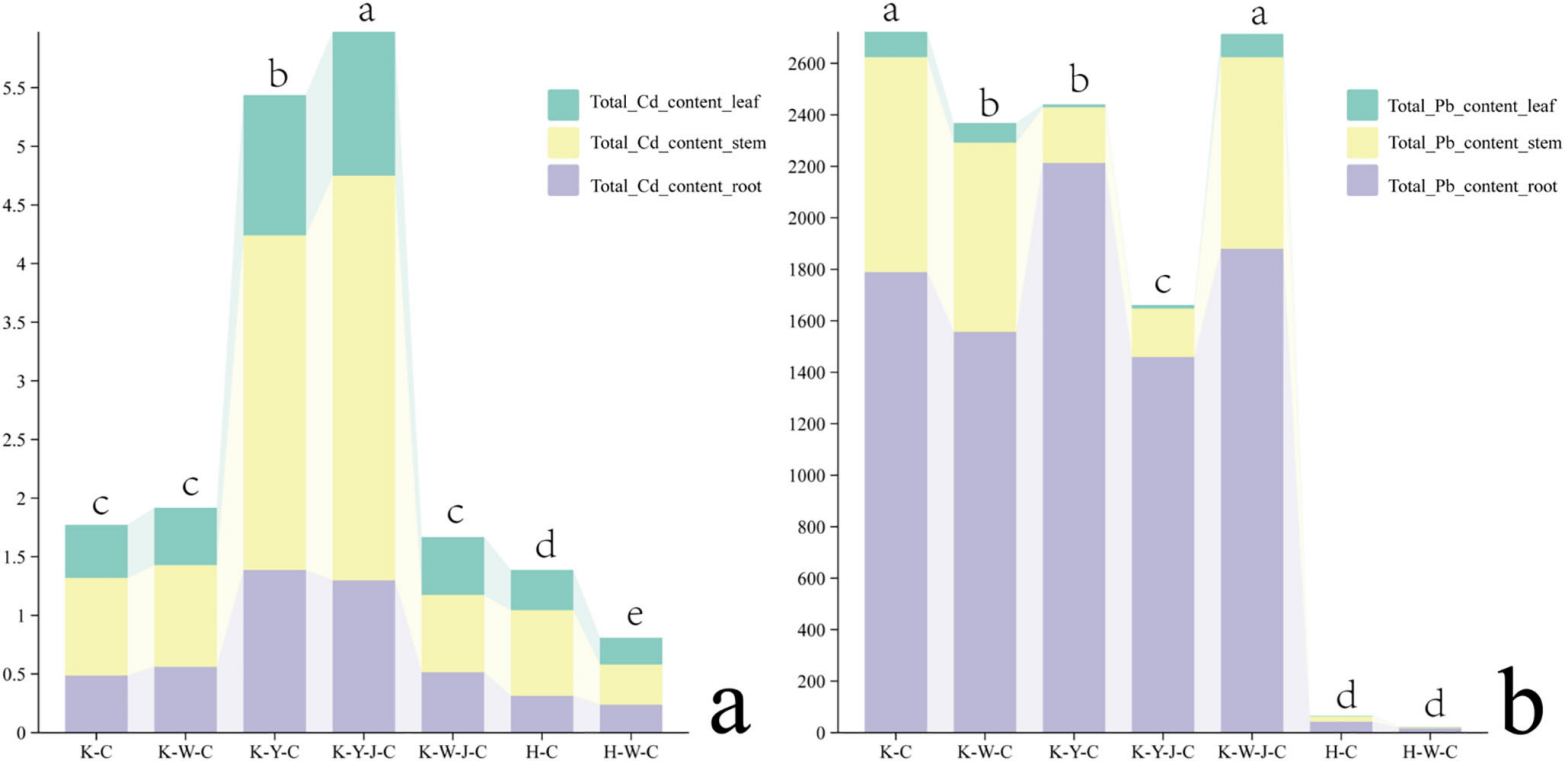
Cd and Pb uptake of dry matter in roots, stems and leaves among samples. **(A)** (Cd uptake of dry matter in roots, stems and leaves among samples), **(B)** (Pb uptake of dry matter in roots, stems and leaves among samples). Content units: 10–3 mg. Mining tailings + *Eucalyptus camaldulensis* (K-C), Mining tailings + Inorganic Fertilizer + *E. camaldulensis* (K-W-C), Mining tailings + Organic Fertilizer + *E. camaldulensis* (K-Y-C), Mining tailings + Organic Fertilizer + *Bacillus subtilis* + *E. camaldulensis* (K-Y-J-C), Mining tailings + Inorganic Fertilizer + *B. subtilis* + *E. camaldulensis* (K-W-J-C), Basic soil + *E. camaldulensis* (H-C), and Basic soil + Inorganic Fertilizer + *E. camaldulensis* (H-W-C). Total_Cd_content_leaf, Total Cd content in leaf; Total_Cd_content_stem, Total Cd content in stem; Total_Cd_content_roof, Total Cd content in root; Total_Pb_content_leaf, Total Pb content in leaf; Total_Pb_content_stem, Total Pb content in stem; Total Pb content in leaf; Total_Pb_content_root, Total Pb content in root. Different letters in a row represent significant differences (P < 0.05) by ANOVA test and Duncan multiple comparative analysis.

Upon quantitative analysis of the dry matter, a comparative assessment of the total uptake of Cd and Pb was conducted. Cd predominantly accumulated in the stems of *E. camaldulensis*, whereas Pb accumulated in the roots (**Figure 7**). The uptake of Cd was significantly enhanced with the application of organic fertilizer with further enhancement when bacterial fertilizers were applied in addition (**Figure 7A**). In the context of Pb uptake (**Figure 7B**), both organic and inorganic fertilizers diminish the accumulation of Pb in *E. camaldulensis*. Notably, the combined application of organic and bacterial fertilizers further reduced the Pb uptake (**Figure 7B**).

## 4 Discussion

The soil in regions affected by mining activities is frequently subjected to severe contamination by a diverse and complex array of heavy metals, with varying types and concentrations (Ren et al., 2022). Historically, research on heavy metal stress in plants has primarily focused on individual heavy metal exposures within controlled laboratory conditions, often neglecting the complex interactions of multiple stressors present in actual mining environments (Talebzadeh, 2023; Zhang S. et al., 2023). *E. camaldulensis* has emerged as a promising candidate for the phytoremediation of metal pollution in mining areas, as evidenced by recent studies (Sadiq et al., 2023; Shirazi et al., 2023). This study contributes to the body of knowledge by examining the efficacy of phytoremediation strategies under conditions that more closely resemble real-world mining scenarios. The findings indicate that the application of organic and bacterial fertilizers, specifically K-Y-J-C, can significantly mitigate and, in some cases, negate the adverse effects of heavy metal stress on *E. camaldulensis*. These results provide valuable insights and open up new avenues for the management of heavy metal pollution in mining regions.

In addition to the accumulation of Pb and Cd in the tissues of the roots, stems, and leaves, our study has revealed that the exposure of *E. camaldulensis* to mining tailings (K-C) primarily resulted in a reduction of Ca, P, K, and the dry weight of both the stems and roots. Furthermore, an increase in the concentrations of chelating agents (CA) and secretory siderophores within the root tissues was observed. It has been noted that trees not subjected to heavy metal stress, denoted as H-C and H-W-C, exhibited lower levels of metal contamination and enhanced growth. The application of fertilizers, as evaluated in this study, has demonstrated a significant enhancement in the heavy metal stress tolerance of *E. camaldulensis*. Specifically, the organic fertilizers (K-Y-C and K-Y-J-C) have been found to be more effective than their inorganic counterparts (K-W-C and K-W-J-C) in improving the plant’s resilience. Moreover, the synergistic application of bacterial fertilizers has been observed to augment the efficacy of the organic fertilizers, thereby further fortifying the plant’s adaptability to metal stress. It is essential to underscore the importance of these findings in the context of environmental remediation and sustainable agriculture, as they provide insights into the potential of specific fertilizers to mitigate the detrimental effects of heavy metal contamination on plant health.

**Figure 8:**
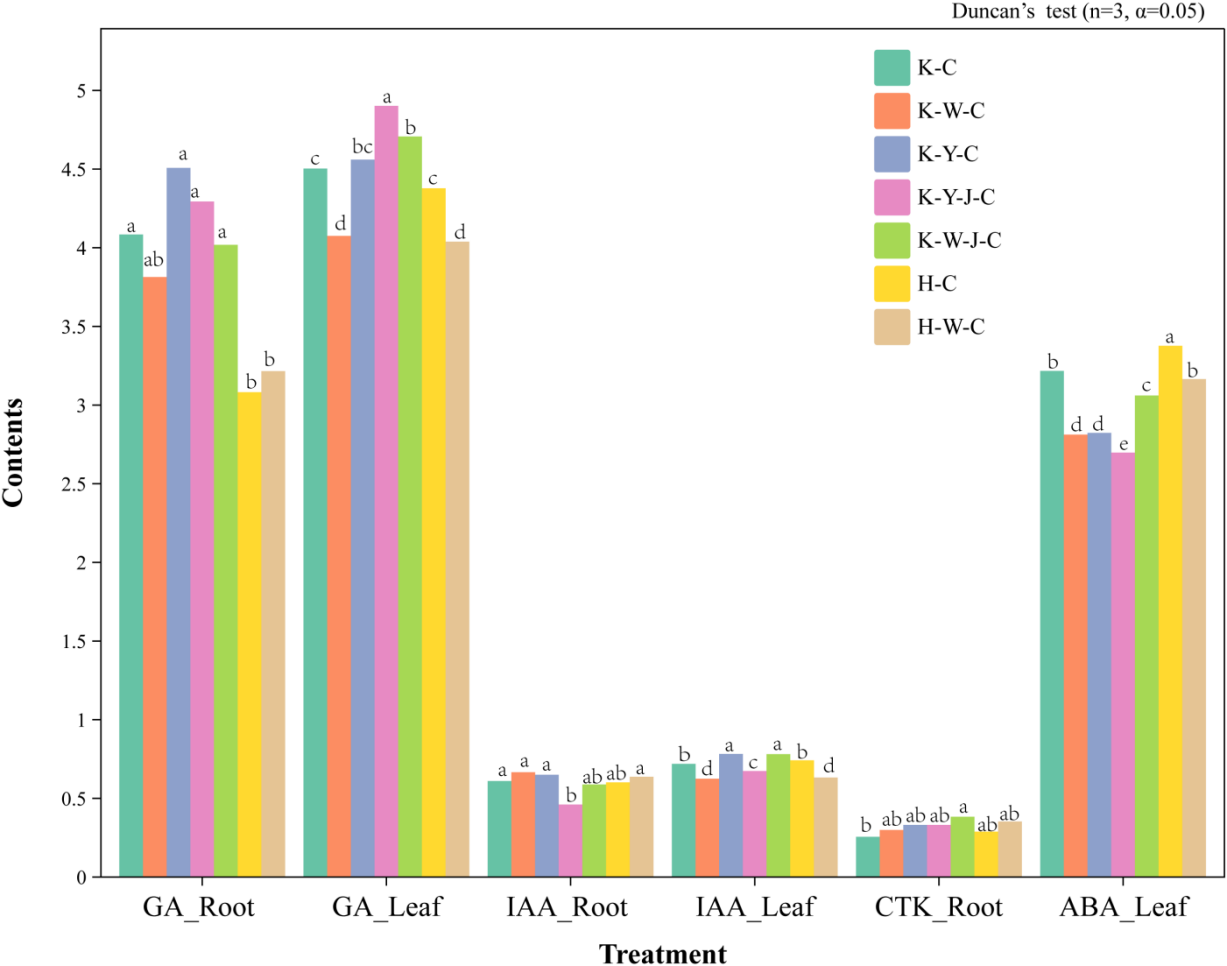
Changes in hormone contents in roots, stems, and leaves among samples. Content units: GA ng/g; IAA μg/g; CTK μg/g; ABA μg/g. Mining tailings + *Eucalyptus camaldulensis* (K-C), Mining tailings + Inorganic Fertilizer + *E. camaldulensis* (K-W-C), Mining tailings + Organic Fertilizer + *E. camaldulensis* (K-Y-C), Mining tailings + Organic Fertilizer + *Bacillus subtilis* + *E. camaldulensis* (K-Y-J-C), Mining tailings + Inorganic Fertilizer + *B. subtilis* + *E. camaldulensis* (K-W-J-C), Basic soil + *E. camaldulensis* (H-C), and Basic soil + Inorganic Fertilizer + *E. camaldulensis* (H-W-C). GA, Gibberellins; IAA, Indole acetic acid; CTK, Cytokinin; ABA, Abscisic acid.

Inorganic fertilizers (H-W-C, K-W-C, and K-W-J-C) increased root K, P (**Figure 4**), and GPX contents (**Figure 3A**). It decreased root OA (**Figure 4**) and leaf ABA (**Figure 8**). K plays a pivotal role in carbohydrate synthesis, transportation, and transformation, which can further promote the development of tree mechanical tissues and tolerance (Chambi-Legoas et al., 2023). P can enhance tree vitality, stimulate the growth of new roots, increase absorption capacity, facilitate the uptake of potassium, and improve tree tolerance (Yao et al., 2023). GPX is known to enhance plant resistance under heavy metal stress (Bela et al., 2015; Sugimoto et al., 1997). The decrease of OA content in roots could be attributed to the secretion of these compounds to the soil by the roots, which may alter the rhizosphere soil environment. This subsequently promotes adaptability to heavy metal stress and enhances heavy metal element uptake (Tindanzor et al., 2023; Zhao et al., 2023). In response to Cd stress, ABA may synergistically induce water stress with Cd (Rehman et al., 2022). K-W-C and K-W-J-C may have increased the GPX content in the roots and the uptake of K and P and also decreased root OA and leaf ABA. These improve plant development and soil conditions while mitigating water stress (Chen et al., 2023). This result was similar to the conclusion that inorganic fertilizer could alleviate heavy metal stress in oil crops (Rizwan et al., 2017). Compared with inorganic fertilizer, the application of organic fertilizer (K-Y-C and K-Y-J-C) increased the growth of plants (ground diameter and height) (**Figure 2**) and the concentration of Ca in roots and decreased the concentration of Cd in roots and concentration of Pb in the whole plant (**Figure 4**). Ca functioning as a signaling molecule may promote the development of young roots and diameters as well as root-hair formation. Ca also helps to preserve the functional structure of cell membranes and increase protoplasm viscosity to improve stress tolerance (An et al., 2023). The result of this study indicates that organic fertilizer can more effectively enhance root development and plant growth, thus reducing the concentration of toxic substances by increasing the biomass of *E. camaldulensis*. This result complies with the finding that organic amendments could promote the growth of energy plants (poplar, willow, and miscanthus) and increase heavy metal tolerance (Al-Lami et al., 2022). In addition, the combined application of organic fertilizer and bacteria increased the content of Cd in the stems and leaves, while Pb was enriched in the roots (**Figure 7**). It may also be related to the different interaction mechanisms of *B. subtilis* on Cd and Pb. This result is consistent with the discovery that *B. subtilis* could activate Cd and cure Pb in tailings improved by organic fertilizer (Yang et al., 2024). The activated Cd is easier to uptake, and the solidified Pb is more difficult to uptake. Intriguingly, compared with untreated tailings, the effects of combined organic and bacterial fertilizer on the transport of Cd and Pb were inconsistent, indicating that the uptake and transport mechanisms of Cd and Pb in *E. camaldulensis* may be different, and further research is needed. It was hypothesized that Cd and Pb may interfere with Ca transporters (Mobin and Khan, 2007) and deter ground diameter expansion.

The effect of bacterial fertilizer is intriguing in this study. When applied in conjunction with inorganic fertilizer (K-W-J-C), it had minimal or even detrimental effects. This result was contrary to the conclusion that the combined application of *B. subtilis* and urea can increase corn yield (Li et al., 2019) and that the combined application of molybdenum fertilizer and *B. subtilis* can increase cabbage yield (Ma et al., 2022). It may be caused by competition between bacterial fertilizer and *E. camaldulensis* for soil nutrition, particularly Ca, during growth in an extremely nutritionally poor environment of tailings (Han et al., 2020; Yang and Zhang, 2022). However, bacterial fertilizers exhibited a synergistic effect when applied with organic fertilizer (K-Y-J-C) possibly because the organic material in the fertilizer plays a key role in the growth of *E. camaldulensis* and the efficacy of the bacterial fertilizer (Shi et al., 2023). This result was consistent with the conclusion that adding *B. subtilis* can further enhance the beneficial effect of leguminous bio-compound manure on plant growth and soil restoration (Abdelfattah et al., 2021).

## 5 Conclusion

Heavy metal stress induced by exposure to mining tailings predominantly impacts the radial growth and biomass accumulation of *E. camaldulensis* by disrupting the uptake of essential nutrients such as K, P, and Ca. This stress predominantly affects the ground diameter and dry weight, with minimal influence on the height of the tree. Pb is predominantly accumulated in the roots, while Cd is primarily localized in the stems of *E. camaldulensis*. The application of organic fertilizer ameliorates stress tolerance and mitigates the adverse effects of heavy metal stress on *E. camaldulensis*. Notably, the concurrent application of bacterial and organic fertilizers yielded more efficacious outcomes. Furthermore, this combined application of different fertilizers enhances the plant’s enrichment capacity for Cd and its tolerance to Pb. The improved heavy metal tolerance in *E. camaldulensis* is likely due to increased uptake of K, P, and Ca, as well as elevated levels of GPX and GA in roots, consequent to the use of organic fertilizers. The principal role of bacterial fertilizer is therefore suggested to cause the reduction of root-borne secretions of OA and CA. The upward translocation of Cd and Pb may be modulated by the root concentrations of OA and CA. It is hypothesized that Cd and Pb interfere with Ca transport mechanisms, thereby exerting a significant impact on the growth of the ground diameter.

## Data Availability

The original contributions presented in the study are included in the article/supplementary material. Further inquiries can be directed to the corresponding authors.
